# Absence of Obesity Paradox in All-Cause Mortality Among Chinese Patients With an Implantable Cardioverter Defibrillator: A Multicenter Cohort Study

**DOI:** 10.3389/fcvm.2021.730368

**Published:** 2021-12-03

**Authors:** Bin Zhou, Xuerong Sun, Na Yu, Shuang Zhao, Keping Chen, Wei Hua, Yangang Su, Jiefu Yang, Zhaoguang Liang, Wei Xu, Min Tang, Shu Zhang

**Affiliations:** ^1^Laboratory of Heart Center, Department of Cardiology, Zhujiang Hospital, Southern Medical University, Guangzhou, China; ^2^Arrhythmia Centre, National Centre for Cardiovascular Diseases, National Clinical Research Center of Cardiovascular Diseases, State Key Laboratory of Cardiovascular Disease, Fuwai Hospital, Chinese Academy of Medical Sciences and Peking Union Medical College, Beijing, China; ^3^Key Laboratory of Endocrinology of National Health Commission, Department of Endocrinology, Peking Union Medical College Hospital, Chinese Academy of Medical Sciences and Peking Union Medical College, Beijing, China; ^4^Department of Cardiology, Shanghai Institute of Cardiovascular Diseases, Zhongshan Hospital, Fudan University, Shanghai, China; ^5^Department of Cardiology, Beijing Hospital, Beijing, China; ^6^Department of Cardiology, First Affiliated Hospital of Harbin Medical University, Harbin, China; ^7^Department of Cardiology, Nanjing Drum Tower Hospital, Nanjing, China

**Keywords:** obesity paradox, body mass index, all-cause mortality, implantable cardioverter-defibrillator, Chinese

## Abstract

**Background:** The results of studies on the obesity paradox in all-cause mortality are inconsistent in patients equipped with an implantable cardioverter-defibrillator (ICD). There is a lack of relevant studies on Chinese populations with large sample size. This study aimed to investigate whether the obesity paradox in all-cause mortality is present among the Chinese population with an ICD.

**Methods:** We conducted a retrospective analysis of multicenter data from the Study of Home Monitoring System Safety and Efficacy in Cardiac Implantable Electronic Device–implanted Patients (SUMMIT) registry in China. The outcome was all-cause mortality. The Kaplan–Meier curves, Cox proportional hazards models, and smooth curve fitting were used to investigate the association between body mass index (BMI) and all-cause mortality.

**Results:** After inclusion and exclusion criteria, 970 patients with an ICD were enrolled. After a median follow-up of 5 years (interquartile, 4.1–6.0 years), in 213 (22.0%) patients occurred all-cause mortality. According to the Kaplan–Meier curves and multivariate Cox proportional hazards models, BMI had no significant impact on all-cause mortality, whether as a continuous variable or a categorical variable classified by various BMI categorization criteria. The fully adjusted smoothed curve fit showed a linear relationship between BMI and all-cause mortality (*p*-value of 0.14 for the non-linearity test), with the curve showing no statistically significant association between BMI and all-cause mortality [per 1 kg/m^2^ increase in BMI, hazard ratio (*HR*) 0.97, 95% *CI* 0.93–1.02, *p* = 0.2644].

**Conclusions:** The obesity paradox in all-cause mortality was absent in the Chinese patients with an ICD. Prospective studies are needed to further explore this phenomenon.

## Introduction

Being overweight or obese is a global health problem, with almost two-thirds of American adults experiencing overweight or obesity (1) and the latest epidemiological survey data from China show that the proportion of adults in China who are overweight and obese is 28.1 and 5.2%, respectively (2). Being overweight or obese can promote inflammatory responses, cardiac hypertrophy, and fibrosis, which can lead to an increased incidence of cardiovascular disease (CVD) and is associated with numerous adverse CVD prognostic events (3–5). Although obesity is a risk factor for CVD, a phenomenon known as the “obesity paradox” has been identified. “Obesity paradox” means that patients who have already suffered from many types of CVD may have a better prognosis if they are classified as overweight or obese (6). The obesity paradox in all-cause mortality has been identified when better survival is seen in people with higher body mass index (BMI) among the patients with hypertension, coronary heart disease, atrial fibrillation, and heart failure (3–5, 7–13).

For patients equipped with an implantable cardioverter defibrillator (ICD), the results of studies on the obesity paradox in all-cause mortality are inconsistent (14–17). Moreover, the previous studies have focused on European and American populations, and there is a lack of studies on the Chinese population with large sample size. This study retrospectively analyzed multicenter data from China to investigate whether the obesity paradox all-cause mortality is present in the Chinese population with an ICD.

## Methods

### Study Population

**Figure 1:**
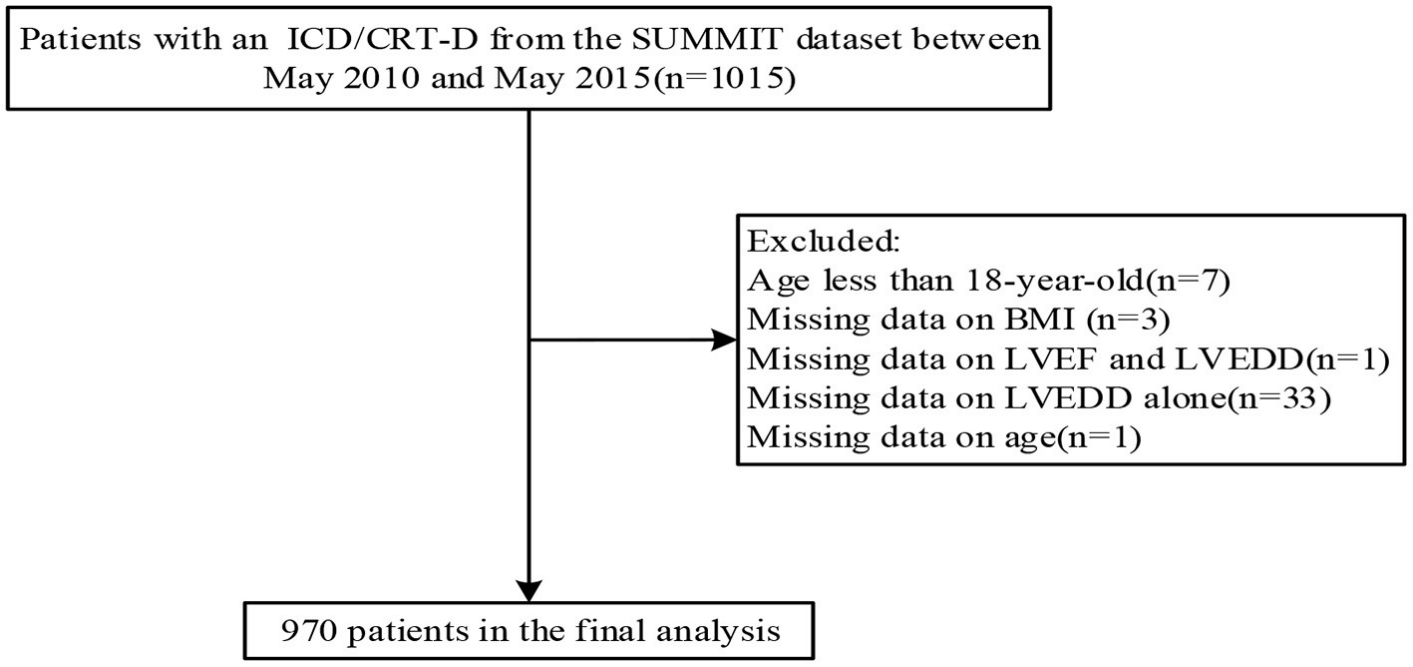
Flowchart of the study population. Abbreviations are shown in Table 1.

Based on data from the Study of Home Monitoring System Safety and Efficacy in Cardiac Implantable Electronic Device–implanted Patients (SUMMIT) registry, we conducted a retrospective cohort analysis enrolling the patients between May 2010 and May 2015 in China. Inclusion criteria were as follows: (i) patients aged more than 18 years; (ii) patients met indications of primary or secondary prevention of sudden cardiac death (SCD) according to clinical practice standards (18–20); and (iii) patients were implanted with an ICD or cardiac resynchronization therapy defibrillator (CRT-D) (collectively referred to as ICD) (Biotronik, Germany) device with home monitoring (HM). Exclusion criteria were as follows: (i) patients under the age of 18 years and (ii) patients with missing clinical data. Figure 1 depicts the flowchart of the research population. The study protocols were approved by the Ethics Committee of Fuwai Hospital, the Chinese Academy of Medical Sciences (the lead institute), and all other collaborating organizations (Zhongshan Hospital, Fudan University, and so on). The protocols followed the Declaration of Helsinki. Before the registry, all patients signed informed permission forms. Strengthening the Reporting of Observational Studies in Epidemiology (STROBE) principles were followed for all reporting (21).

### Data Collection

The BMI was determined by dividing the patient's weight (kg) by the square of his or her height (m^2^), and the result was expressed as kg/m^2^. BMI grouped according to tertiles, WHO criteria (22), Asian criteria (23), or Chinese criteria (24). Before ICD implantation, baseline clinical information was obtained from the patients' medical records.

### Device Programming Settings and Outcome

The device programming settings technique was the same as in our prior study (25), which was described in detail in Supplementary Material. The outcome was all-cause mortality. Routine follow-ups were undertaken by phone calls and outpatient clinic visits. In the case that the transmission of their data was disrupted, and patient status was confirmed *via* phone calls. The last follow-up visit was in June 2018.

### Statistical Analysis

The means ± SD or proportions are used to present the data. To compare the all-cause mortality of different BMI groups, we used Kaplan–Meier curves (log-rank test). A Cox proportional hazards model was used to furtherly assess the association between BMI and all-cause mortality. A univariate Cox proportional hazards model was first used to explore the factors influencing all-cause mortality. The multivariate Cox proportional hazards models were conducted by adjusting for factors that had a statistically significant effect on death at the 0.05 level in the univariate Cox model or all baseline factors. To further explore the dose-response effect of BMI on all-cause mortality, a cubic spline function model and smooth curve fitting (penalized spline method) were conducted. To ensure the robustness of the result, we did the following sensitivity analysis. First, we converted the BMI into a categorical variable by tertiles and calculated the *P* for trend. Second, we performed the same analysis based on WHO criteria, Asian criteria, or Chinese criteria for BMI grouping. Third, we did subgroup analyses in various groups. To maximize the exploration of other possible risk factors for all-cause mortality, the univariate and multivariate Cox models were used to do the analysis. R version 4.0.0 (R Foundation for Statistical Computing, Vienna, Austria) and Empower (R) were used for all the analyses (X&Y Solutions, Inc., Boston, MA, USA). All *P*-values were considered statistically significant if they were <0.05 (two-sided).

## Results

### Baseline Characteristics of the Study Population

A total of 1,015 patients from the SUMMIT dataset between May 2010 and May 2015 were initially included. After inclusion and exclusion criteria, 970 patients were enrolled. Table 1 shows the overall baseline characteristics of the study population. The average age of the study population was 60.3 years, with 72.9% male. The percentage of New York Heart Association [NYHA] class III/IV was 49.9 and 27.4% of patients were implanted with CRT-D. In total, 394 patients met the secondary prevention of SCD criteria. Among these patients, 236 (60%) had documented sustained ventricular tachycardia (VT), 98 (25%) had documented ventricular fibrillation (VF) and resuscitated SCD, and 60 (15%) experienced unexplained syncope and may be induced to VT or VF during the electrophysiological study. We performed a comparison of baseline characteristics based on the Chinese grouping criteria for BMI. Most of the variables were not significantly different, except that the obese population had a higher proportion of men, higher systolic and diastolic blood pressure, a greater proportion of ischemic cardiomyopathy, a higher proportion of hypertension, and a greater history of stroke (all *P* < 0.05).

**Table 1:** Baseline characteristics of study population.

**Variables**	**Total**	**BMI <24**	**BMI: 24–28**	**BM ≥ 28**	***p***
		**kg/m^**2**^**	**kg/m^**2**^**	**kg/m^**2**^**	
	**(*n* = 970)**	**(*n* = 567)**	**(*n* = 352)**	**(*n* = 51)**	
Male, *n* (%)	707 (72.9)	392 (69.1)	275 (78.1)	40 (78.4)	0.008
Age at implantation, years	60.3 ± 13.5	60.3 ± 13.7	60.7 ± 13.0	58.3 ± 15.0	0.498
NYHA, Class III/IV, *n* (%)	484 (49.9)	288 (50.8)	170 (48.3)	26 (51)	0.753
SBP, mmHg	124.5 ± 17.4	123.5 ± 17.1	125.0 ± 17.3	131.9 ± 19.9	0.004
DBP, mmHg	76.9 ± 10.9	76.1 ± 10.6	77.5 ± 10.7	80.7 ± 13.8	0.005
Primary prevention, *n* (%)	576 (59.4)	343 (60.5)	199 (56.5)	34 (66.7)	0.273
CRT-D, *n* (%)	266 (27.4)	163 (28.7)	90 (25.6)	13 (25.5)	0.548
Ischemic cardiomyopathy, *n* (%)	324 (33.4)	174 (30.7)	127 (36.1)	23 (45.1)	0.046
Dilated cardiomyopathy, *n* (%)	238 (24.5)	142 (25)	84 (23.9)	12 (23.5)	0.908
Hypertrophic cardiomyopathy, *n* (%)	37 (3.8)	19 (3.4)	13 (3.7)	5 (9.8)	0.091
Long QT syndrome, *n* (%)	12 (1.2)	7 (1.2)	5 (1.4)	0 (0)	0.888
Hypertension, *n* (%)	305 (31.4)	163 (28.7)	114 (32.4)	28 (54.9)	<0.001
Diabetes mellitus, *n* (%)	101 (10.4)	51 (9)	42 (11.9)	8 (15.7)	0.164
Stroke, *n* (%)	18 (1.9)	5 (0.9)	10 (2.8)	3 (5.9)	0.01
Atrial fibrillation, *n* (%)	104 (10.7)	60 (10.6)	38 (10.8)	6 (11.8)	0.965
Pre-implant syncope, *n* (%)	194 (20.0)	114 (20.1)	78 (22.2)	2 (3.9)	0.01
LVEF, %	42.5 ± 14.9	42.1 ± 15.0	42.8 ± 14.7	44.4 ± 15.5	0.503
LVEDD, mm	58.8 ± 13.1	58.4 ± 13.2	59.7 ± 13.0	57.3 ± 13.3	0.252
β-Blocker, *n* (%)	566 (58.4)	326 (57.5)	209 (59.4)	31 (60.8)	0.8
Amiodarone, *n* (%)	290 (29.9)	171 (30.2)	109 (31)	10 (19.6)	0.248
ACE or ARB, *n* (%)	360 (37.1)	207 (36.5)	132 (37.5)	21 (41.2)	0.79
Diuretic, *n* (%)	382 (39.4)	212 (37.4)	149 (42.3)	21 (41.2)	0.318
Loop diuretic, *n* (%)	280 (28.9)	159 (28)	106 (30.1)	15 (29.4)	0.794
Aldosterone antagonist, *n* (%)	363 (37.4)	210 (37)	138 (39.2)	15 (29.4)	0.385

*ACEI, angiotensin-converting enzyme inhibitor; ARB, angiotensin receptor blocker; BMI, body mass index; CRT-D, cardiac resynchronization therapy defibrillator; DBP, diastolic blood pressure; LVEF, left ventricular ejection fraction; LVEDD, left ventricular end-systolic dimension; NYHA, New York Heart Association; SBP, systolic blood pressure*.

### Influence of BMI on All-Cause Mortality

**Figure 2:**
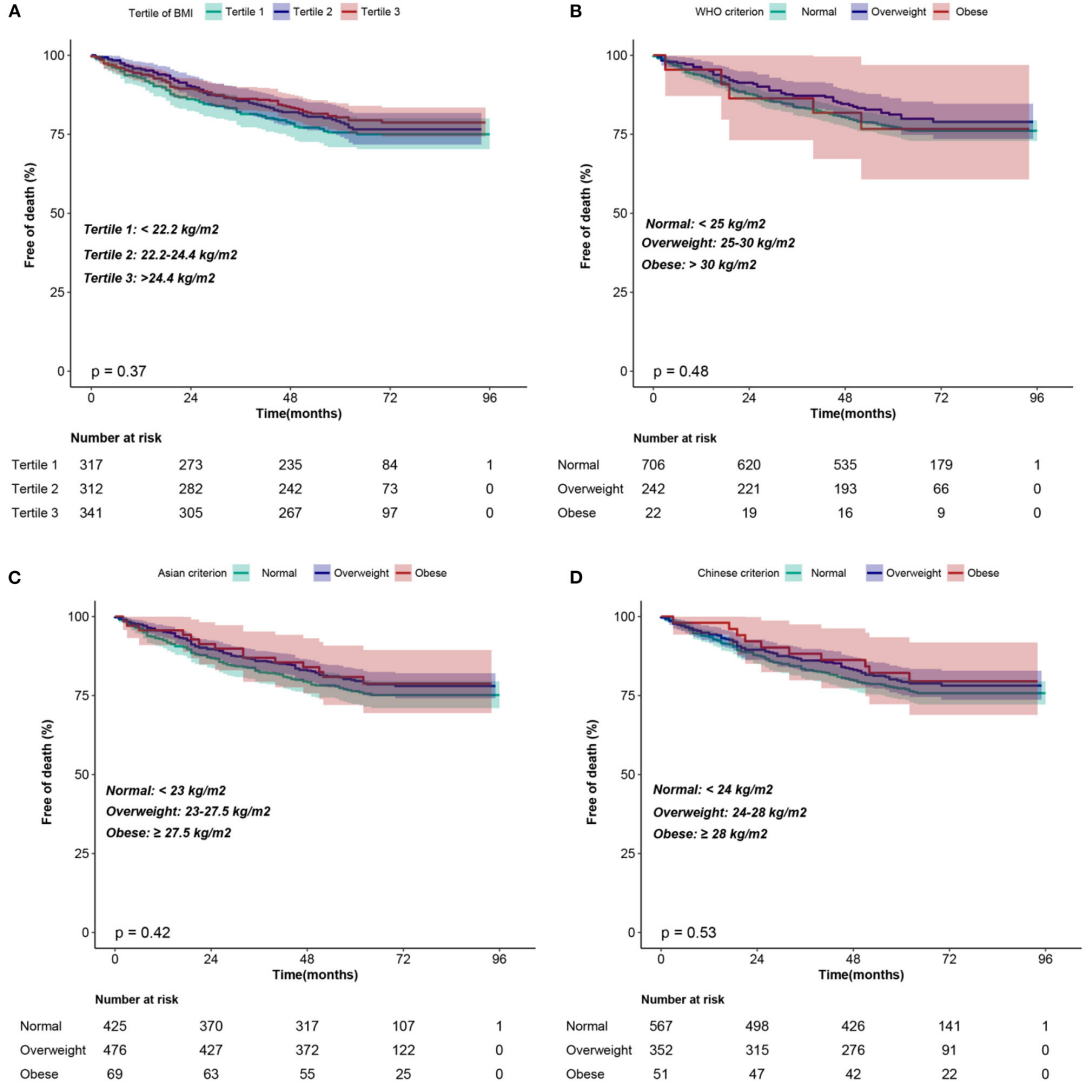
Kaplan–Meier curves of the cumulative probability of survival according to the BMI classification of **(A)** tertiles, **(B)** WHO criterion, **(C)** Asian criterion, **(D)** Chinese criterion. BMI, body mass index.

The median follow-up was 5.0 years (interquartile, 4.1–6.0 years). During follow-up, 213 (22.0%) patients experienced all-cause mortality. Kaplan–Meier curves were plotted to compare the cumulative probability of survival for different BMI groups (Figure 2). The cumulative probability of survival did not differ between the groups regardless of the criteria of BMI grouping (log-rank, all *p* > 0.05).

**Figure 3:**
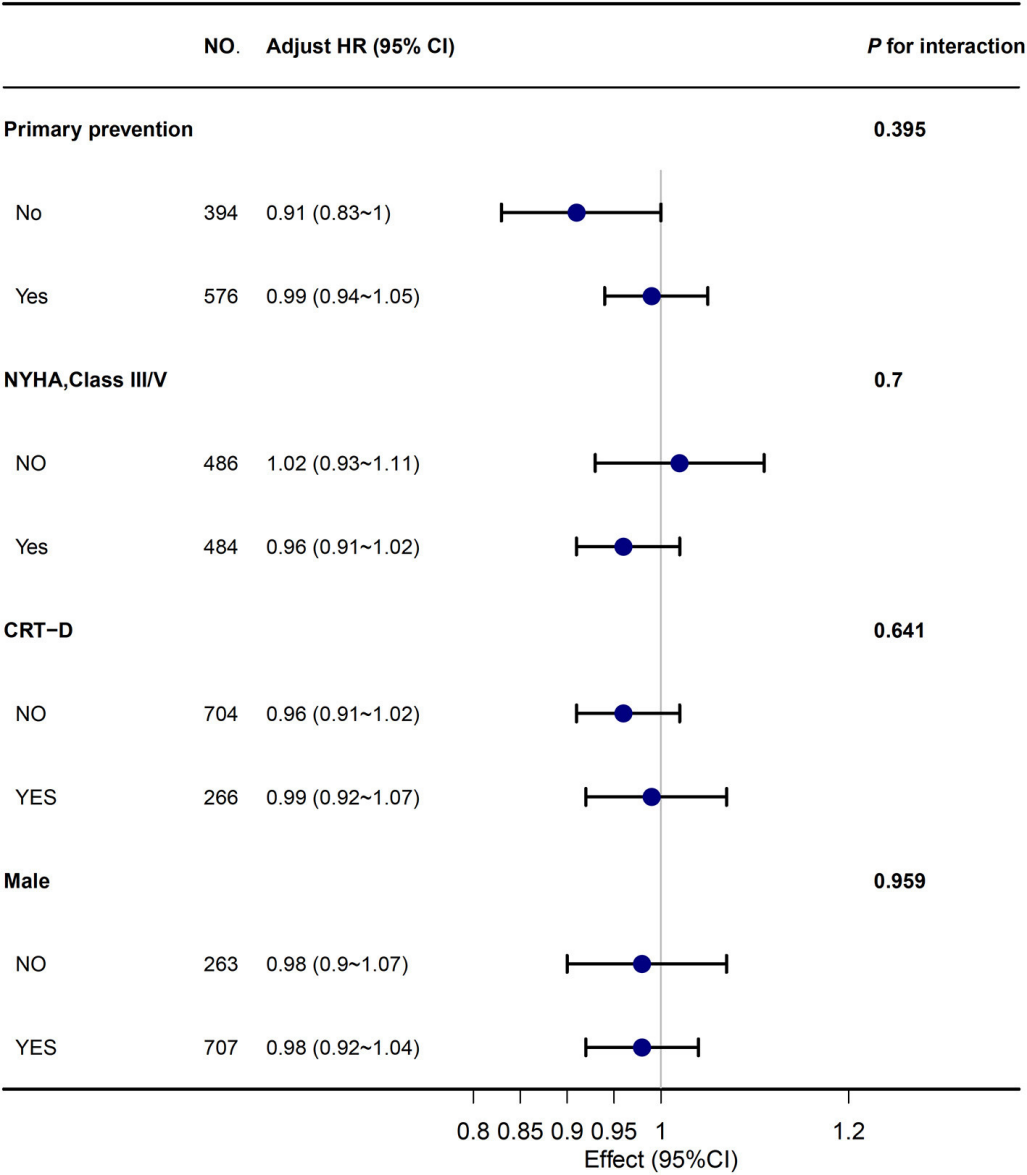
The association between BMI and all-cause mortality in subgroups. Abbreviations are shown in Table 1.

Table 2 shows the effect of BMI on all-cause mortality using four Cox proportional risk models. In the unadjusted model (model 1), each 1 kg/m^2^ increase in BMI was associated with a 2% increase in the risk of death, but this was not statistically significant. Further, in model 2, the association between BMI and death was again not statistically significant after adjusting for age and sex. After adjustment for additional covariates in model 3 (for age, New York Classification of Cardiac Function (NYHA) III/IV, primary prevention, ischaemic cardiomyopathy, hypertension, diabetes, atrial fibrillation, left ventricular ejection fraction (LVEF), left ventricular end-systolic dimension (LVEDD), β-blocker, angiotensin-converting enzyme inhibitor (ACEI)/angiotensin receptor blocker (ARB), a loop diuretic, aldosterone antagonist, and dilated cardiomyopathy) and all covariates in Table 1 in model 4, the results underwent negligible changes. In addition, we converted BMI from a continuous variable to a categorical variable. There was no significant increase in the risk of death in patients with tertile 2 or tertile 3 in all models (models 1–4) compared with tertile 1 as a reference. The test for trend was not significant in any of the models. In addition, the sensitivity analyses were performed using different clinical classification criteria for BMI and the results remained consistent. Additionally, we analyzed different subgroups and the results were still robust (Figure 3).

**Table 2:** Association of BMI with all-cause mortality in different Cox proportional hazards models.

**BMI (kg/m^**2**^)**		**Model 1**		**Model 2**		**Model 3**		**Model 4**	
	**No. of death**	**HR (95% CI)**	**P-value**	**HR (95% CI)**	**P-value**	**HR (95% CI)**	**P-value**	**HR (95% CI)**	***P*** **-value**
**Continuous**	213	0.98 (0.94, 1.03)	0.4405	0.98 (0.93, 1.02)	0.3507	0.97 (0.92, 1.02)	0.1937	0.97 (0.93, 1.02)	0.2644
**Tertiles**									
<22.1	96	Reference		Reference		Reference		Reference	
22.1–24.4	126	0.87 (0.63, 1.20)	0.4002	0.86 (0.62, 1.19)	0.3560	0.89 (0.64, 1.25)	0.5163	0.89 (0.63, 1.25)	0.4949
> 24.4	130	0.79 (0.57, 1.10)	0.4405	0.77 (0.55, 1.07)	0.1175	0.72 (0.51, 1.01)	0.0571	0.73 (0.52, 1.04)	0.0793
*P _*trend*_-value*			0.1645		0.1178		0.0560		0.0791
**WHO criterion**									
<25	245	Reference		Reference		Reference		Reference	
25–30	100	0.82 (0.59, 1.13)	0.2313	0.80 (0.57, 1.10)	0.1692	0.72 (0.52, 1.01)	0.0570	0.76 (0.54, 1.06)	0.1048
≥30	7	0.99 (0.40, 2.40)	0.9757	1.02 (0.42, 2.49)	0.9585	0.89 (0.36, 2.20)	0.8004	0.99 (0.40, 2.40)	0.9757
*P _*trend*_-value*			0.3221		0.2701		0.0941		0.1296
**Asian criterion**									
<23	138	Reference		Reference		Reference		Reference	
23–27.5	183	0.84 (0.64, 1.11)	0.2186	0.82 (0.62, 1.08)	0.1583	0.79 (0.59, 1.06)	0.1129	0.84 (0.64, 1.11)	0.2186
≥ 27.5	31	0.81 (0.46, 1.42)	0.4679	0.77 (0.44, 1.34)	0.3515	0.71 (0.40, 1.26)	0.2433	0.72 (0.40, 1.29)	0.2726
*P _*trend*_-value*			0.2181		0.1420		0.0870		0.0968
**Chinese criterion**									
<24	197	Reference		Reference		Reference		Reference	
24–28	131	0.87 (0.65, 1.16)	0.3286	0.84 (0.63, 1.12)	0.2364	0.79 (0.59, 1.06)	0.1105	0.80 (0.60, 1.08)	0.1520
≥ 28	24	0.80 (0.42, 1.52)	0.4888	0.79 (0.42, 1.50)	0.4739	0.72 (0.38, 1.39)	0.3335	0.73 (0.38, 1.42)	0.3574
*P _*trend*_-value*			0.2694		0.2074		0.0901		0.1219

*Model 1: adjusted for none. Model 2: adjusted for age, gender. Model 3: adjusted for variables in Model 2 plus NYHA, Class III/IV, primary prevention, Ischemic cardiomyopathy, hypertension, diabetes mellitus, atrial fibrillation, LVEF, LVEDD. β-Blocker, ACEI or ARB, a loop diuretic, aldosterone antagonists, dilated cardiomyopathy. Model 4 adjusted for all covariates presented in Table 1. HR, hazard ratio; other abbreviations are shown in Table 1*.

**Figure 4:**
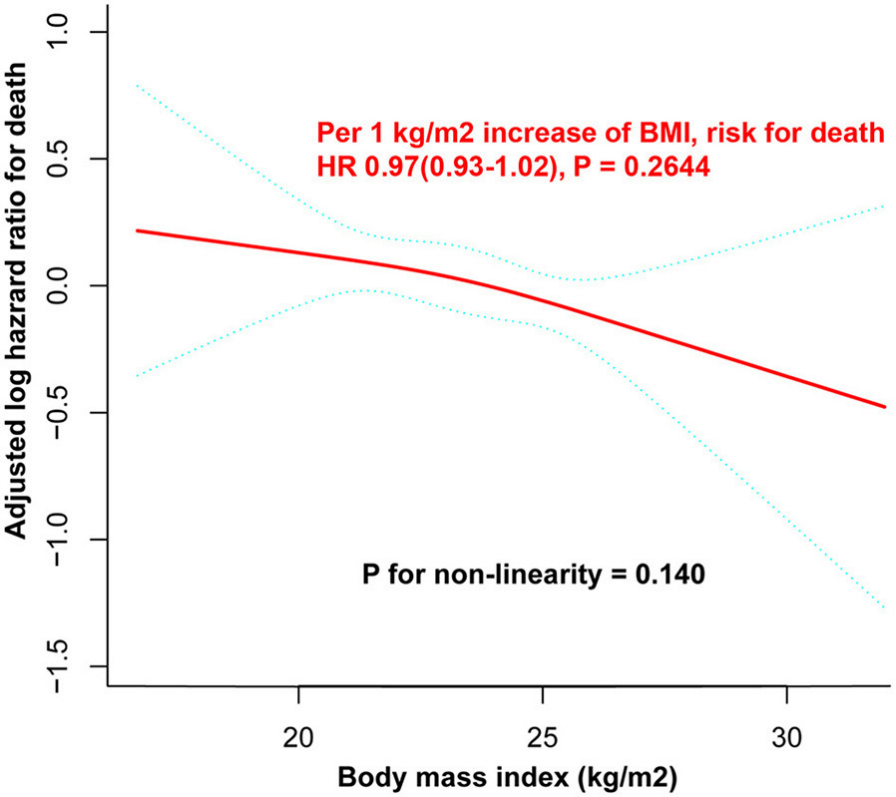
Dose-response curve between BMI and all-cause mortality. There was a linear relationship between BMI and all-cause mortality (*P* for non-linearity = 0.140). The adjusted log *HR* and its 95% *CI* are represented by the solid blue and dashed blue lines, respectively. All the covariates listed in Table 1 were used as adjustment factors. No statistically significant association between BMI and all-cause mortality was observed (for every increase of 1 kg/m^2^ BMI, *HR* 0.97, 95% *CI* 0.93–1.02, *P* = 0.2644). BMI, body mass index; CI, confidence interval; HR, hazard ratio.

A cubic spline function model and smoothed curve fitting (penalized spline approach) were performed to assess the dose-response association between BMI and all-cause mortality. The fully adjusted smoothed curve fit showed a linear relationship between BMI and mortality (*p*-value of 0.14 for the non-linearity test) (Figure 4), with the curve showing no statistically significant association between BMI and all-cause mortality (per 1 kg/m^2^ increase in BMI, hazard ratio (*HR*) 0.97, 95% *CI* 0.93–1.02, *p* = 0.2644).

### Univariate and Multivariate Risk Factors of All-Cause Mortality

Table 3 shows the univariate Cox proportional hazards models of all-cause mortality. Oder age, NYHA class III/IV, primary prevention, ischaemic cardiomyopathy, hypertension, diabetes, atrial fibrillation, lower LVEF, wider LVEDD, β-blocker, ACEI/ARB, a loop diuretic, aldosterone antagonist, and dilated cardiomyopathy were the univariate predictors of all-cause mortality in the overall group. Older age (*HR* 1.02; 95% *CI* 1.01–1.04; *P* < 0.001), NYHA Class III/V (*HR* 1.55; 95% *CI* 1.1–2.19; *P* < 0.012), ischemic cardiomyopathy (*HR* 1.54; 95% *CI* 1.14–2.07; *P* < 0.005), wider LVEDD (*HR* 1.02; 95% *CI* 1.01–1.04; *p* = 0.01) were independent predictors of increased all-cause mortality.

**Table 3:** The univariate and multivariate risk factors of all-cause mortality.

**Variable**	**Crude model**		Adjust model^*^	
	**HR (95% CI)**	***P*** **-value**	**HR (95% CI)**	***P*** **-value**
BMI	0.98 (0.94–1.03)	0.44	0.97 (0.93–1.02)	0.262
Male	1.2 (0.88–1.65)	0.246	1.05 (0.75–1.48)	0.778
Age	1.03 (1.02–1.04)	<0.001	1.02 (1.01–1.04)	<0.001
NYHA,Class III/V	2.49 (1.86–3.32)	<0.001	1.55 (1.1–2.19)	0.012
SBP	0.99 (0.98–1)	0.072	0.99 (0.98–1)	0.054
DBP	0.99 (0.98–1.01)	0.398	1 (0.99–1.02)	0.586
Primary prevention	1.41 (1.06–1.87)	0.018	0.88 (0.61–1.27)	0.492
CRT-D	1.59 (1.2–2.1)	0.001	0.9 (0.62–1.31)	0.58
Ischemic cardiomyopathy	1.91 (1.46–2.5)	<0.001	1.54 (1.14–2.07)	0.005
Dilated cardiomyopathy	1.47 (1.1–1.96)	0.01	1.02 (0.73–1.44)	0.897
Hypertrophic cardiomyopathy,	0.46 (0.17–1.23)	0.121	0.87 (0.31–2.42)	0.793
Long QT syndrome	0.33 (0.05–2.36)	0.271	0.61 (0.08–4.5)	0.627
Hypertension	1.7 (1.29–2.23)	<0.001	1.36 (0.99–1.86)	0.054
Diabetes mellitus	1.84 (1.28–2.66)	0.001	1.19 (0.8–1.77)	0.379
Stroke	1.58 (0.7–3.56)	0.27	1.09 (0.46–2.56)	0.852
Atrial fibrillation	1.61 (1.11–2.33)	0.012	1.19 (0.81–1.76)	0.382
Pre-implant syncope	0.84 (0.59–1.19)	0.326	0.95 (0.65–1.39)	0.788
LVEF	0.97 (0.96–0.98)	<0.001	1 (0.98–1.01)	0.609
LVEDD	1.03 (1.02–1.04)	<0.001	1.02 (1.01–1.04)	0.001
β-Blocker	1.46 (1.1–1.94)	0.009	1.28 (0.94–1.73)	0.113
Amiodarone	0.84 (0.62–1.13)	0.253	0.81 (0.58–1.13)	0.212
ACE or ARB	1.43 (1.09–1.87)	0.009	0.92 (0.68–1.25)	0.599
Diuretic	2.01 (1.54–2.64)	<0.001	0.78 (0.42–1.44)	0.43
Loop diuretic	1.83 (1.39–2.41)	<0.001	1.26 (0.8–1.97)	0.324
Aldosterone antagonists	1.95 (1.49–2.55)	<0.001	1.33 (0.84–2.1)	0.229

* *Adjusted for all covariates presented in Table 1 except the independent variable itself. Abbreviations are shown in Tables 1, 2*.

## Discussion

### Major Findings

The following are the main findings of study: (1) according to Kaplan–Meier curves and Cox proportional hazards models, BMI had no significant impact on all-cause mortality in the patients with ICD, whether as a continuous variable or a categorical variable classified by various BMI categorization criteria. (2) A linear relationship between BMI and all-cause mortality was identified with the curve showing no statistically significant association between BMI and all-cause mortality.

### Compared With Previous Studies

This study applied various statistical methods for data analysis, all of which indicated that there was no obesity paradox in the Chinese ICD population. This is consistent with the results of a Spanish study (14). However, another research from the United States reported that low BMI was independently associated with death at 1 year (15). In the Multicenter Automatic Defibrillator Implantation Trial-II (MADIT II) study, a study of a retrospective analysis of patients with left ventricular dysfunction after myocardial infarction, obese patients (BMI ≥ 30 kg/m^2^) had a higher survival rate than non-obese patients (16). Another study in the United States found a greater benefit of higher BMI on survival in patients with ICDs, particularly in the older patients (17). The inconsistent results may be due mainly to differences in demographic characteristics, ethnic groups, sample size, or adjusted covariates. In addition, from the MADIT era (26), the pharmacological treatment and prevention strategies for heart failure optimize over the years (27), which may contribute to a lower rate of all-cause mortality. This may make it harder to observe associations of BMI and all-cause mortality. However, the obesity paradox does not appear to be present in the Chinese ICD population as far as the results of this study are concerned.

### Risk Factors Related to All-Cause Mortality Among Patients With an ICD

Our study found that older age, NYHA Class III/V, ischemic cardiomyopathy, and wider LVEDD were independent predictors of increased all-cause mortality. Compared with the patient in NYHA Class I/II, patients with NYHA Class III/V had a 55% increased risk of all-cause mortality. Higher NYHA Class was reported to be associated with a higher rate of 1-year all-cause mortality (15). Thus, our study extended the above findings to a 5-year follow-up, suggesting that the effect of cardiac function class, a very clinically assessable index, on all-cause mortality can last that long. This suggests that we need to pay more attention to the assessment and management of cardiac function class in patients with ICD. Similarly, we should improve the evaluation and management of patients with advanced age, ischemic cardiomyopathy, and left ventricular enlargement.

### Clinical Implications

This study had some clinical implications. First, it clarified that the obesity paradox was not found in the Chinese with an ICD for the time being, adding evidence from the Chinese population to this controversial topic. Second, it illustrated that risk stratification for all-cause mortality based on baseline BMI was not desirable in the Chinese population with an ICD and that there was no need to focus specifically on the value of baseline BMI for the time being. Third, this study suggested that we should pay more attention to the patients with advanced age, ischemic cardiomyopathy, NYHA Class III/IV, and left ventricular enlargement.

### Strengths and Limitations

The study had the following strengths. First, the study was a multicenter study with a relatively large sample size and good generalizability. Second, the study used multiple statistical methods to maximize the exploration of the relationship between BMI and all-cause mortality. Third, the study used various BMI grouping criteria to enhance the robustness of the results. However, the study had some limitations. First, the study was a retrospective observational study and there was some selection bias. Second, the study did not collect data on the blood tests, ECG, etc. that may have influenced the effect of BMI on mortality. We were unable to adjust for these substantial confounders; therefore, prospective studies that collect more variables are needed for further in-depth study. Third, in our study, we used conventional programming setting otherwise the proposed high-rate therapy and delayed ICD therapy were proposed in the Multicenter Automatic Defibrillator Implantation Trial-Reduce Inappropriate Therapy (MADIT-RIT) study (28). The effect of different programming settings on all-cause mortality should be considered. However, all the patients in our study received the same programming setting. So, the prognostic impact of the programming setting on each individual was close. Fourth, the relatively small number of obese patients in the study limited the generalizability of the findings. Last, in our investigation, we did not gather data on adiposity distribution (waist-to-hip ratio or waist circumference) or body fat percentage which were also indicators for obesity. However, it is undeniable that it is used in a wide range of studies (8, 10, 13–17) as a commonly used and easily accessible indicator. In the future, prospective studies that include larger sample sizes to ensure a balanced sample across groups and collect more indicators that respond to obesity are awaited to better illustrate the obesity paradox of all-cause mortality in the Chinese population with an ICD.

## Conclusions

Using various statistical methods of analysis and different BMI grouping criteria, the obesity paradox in all-cause mortality did not emerge in the Chinese population with an ICD. Prospective studies are still needed to further explore this topic.

## Data Availability Statement

The raw data supporting the conclusions of this article will be made available by the authors, without undue reservation.

## Ethics Statement

The studies involving human participants were reviewed and approved by Ethics Committee of Fuwai Hospital, the Chinese Academy of Medical Sciences (the lead institute), and all other collaborating organizations (Zhongshan Hospital, Fudan University, and so on). The patients/participants provided their written informed consent to participate in this study.

## Author Contributions

The study was conceived and designed by SZhang, MT, and BZ. The ICD was implanted by MT, KC, WH, YS, JY, ZL, and WX. The data were collected by SZhao. The data were analyzed and the manuscript was written by BZ, XS, and NY. The manuscript was revised by SZhang and MT. The final manuscript was read and approved by all authors.

## Funding

This work was supported by the Natural Science Foundation of China (81470466) and the National Science & Technology Pillar Program during the 12th Five-Year Plan Period (2011BAI11B02).

## Conflict of Interest

The authors declare that the research was conducted in the absence of any commercial or financial relationships that could be construed as a potential conflict of interest.

## Publisher's Note

All claims expressed in this article are solely those of the authors and do not necessarily represent those of their affiliated organizations, or those of the publisher, the editors and the reviewers. Any product that may be evaluated in this article, or claim that may be made by its manufacturer, is not guaranteed or endorsed by the publisher.
