# Supplementary material for: Absence of Obesity Paradox in All-Cause Mortality Among Chinese Patients With an Implantable Cardioverter Defibrillator: A Multicenter Cohort Study
Source: Front Cardiovasc Med. 2021 Dec 3;8:730368. doi: 10.3389/fcvm.2021.730368 (PMC8677836; doi:10.3389/fcvm.2021.730368)
Supplement: Supplementary file 1 [file Data_Sheet_1.docx]

Supplementary Material

1. **Supplementary Text S1**

Programming settings were as follows: the basic pacing rate was 40-60 beats per minute (bpm), target VT monitor zone was 140-170 bpm, target VT therapy zone was over 170-210 bpm, and VF zone was over 210 bpm. In VT therapy zone, 2-3 bursts of anti-tachycardia pacing (ATP) were delivered, followed by high-energy shock for persisting episodes. In VF zone, high-energy shock alone was used. The detection interval was 26 beats in VT zone with a 20-beats redetection. And the detection interval was 12 out of 16 beats in VF zone. Other programmable parameters are determined by individual doctors. VT refers to spontaneous ventricular depolarization with a frequency of more than 100 bpm, for 3 or more consecutive times. The width of QRS is usually wider than 120ms. VF refers to the disordered agitation of the ventricle, which leads to the regular and orderly agitation and the disappearance of the systolic and diastolic function of the ventricle. Its electrocardiogram (ECG) is as follows: a constant shift in axis and morphology of the electrogram is accompanied by marked and variable changes in electrogram amplitude.

In our study, ICD was equipped with Biotronik SMART® algorithm which could automatically analyze the waveform and frequency of ECG to distinguish VT/VF and supraventricular tachycardia (including atrial fibrillation, atrial flutter, and sinus tachycardia). Additionally, the tachycardia events could be monitored by the ICD and automatically transmitted to the home monitoring system. Two cardiologists reviewed the intra-cardiac electrograms (IEGM) of tachycardia events in a blinded manner to further confirm the event as VT/VF or supraventricular tachycardia and analyzed the VAs to assess whether the patient received the appropriate ICD therapy. When there was a disagreement on the IEGM reading and ICD therapy, a third cardiologist was responsible for a conclusive opinion.
